# Effect of Atomic Charges on Octanol–Water Partition Coefficient Using Alchemical Free Energy Calculation

**DOI:** 10.3390/molecules23020425

**Published:** 2018-02-15

**Authors:** Koji Ogata, Makoto Hatakeyama, Shinichiro Nakamura

**Affiliations:** RIKEN Innovation Center, Nakamura Laboratory, 2-1 Hirosawa, Wako, Saitama 351-0198, Japan; hatakeyama_xeno@riken.jp (M.H.); snakamura@riken.jp (S.N.)

**Keywords:** MD simulation, thermodynamic integration, solvation environment, atomic charge

## Abstract

The octanol–water partition coefficient (log*P_ow_*) is an important index for measuring solubility, membrane permeability, and bioavailability in the drug discovery field. In this paper, the log*P_ow_* values of 58 compounds were predicted by alchemical free energy calculation using molecular dynamics simulation. In free energy calculations, the atomic charges of the compounds are always fixed. However, they must be recalculated for each solvent. Therefore, three different sets of atomic charges were tested using quantum chemical calculations, taking into account vacuum, octanol, and water environments. The calculated atomic charges in the different environments do not necessarily influence the correlation between calculated and experimentally measured ∆*G_water_* values. The largest correlation coefficient values of the solvation free energy in water and octanol were 0.93 and 0.90, respectively. On the other hand, the correlation coefficient of log*P_ow_* values calculated from free energies, the largest of which was 0.92, was sensitive to the combination of the solvation free energies calculated from the calculated atomic charges. These results reveal that the solvent assumed in the atomic charge calculation is an important factor determining the accuracy of predicted log*P_ow_* values.

## 1. Introduction

The octanol–water partition coefficient (log*P_ow_*) is an index used in drug discovery processes to estimate the solubility, membrane permeability, and bioavailability of compounds [1,2]. Several experimental methods for measuring it have been developed [2,3,4,5,6], but they are time consuming and costly, because experimental methods use the actual compounds, and sometimes, the synthesis of a compound takes a long time. To accelerate the drug discovery process and to reduce its cost, we need a log*P_ow_* measurement method that is easier, faster, and more accurate than the current ones.

Computational methods for predicting the log*P_ow_* value have been developed using various techniques. The methods summing the contributions of a compound’s atoms and fragments (e.g., XLOGP3 [7], KOWWIN [8], etc.) rapidly calculate log*P_ow_* values similar to those obtained experimentally, and have therefore been used in the screening of drug-like compounds [9], making it possible to calculate the log*P_ow_* values for the tens of millions of compounds in databases such as PubChem [10] and ZINC [11].

The log*P_ow_* values of compounds can also be predicted accurately by alchemical free energy calculation techniques using molecular dynamics (MD) simulation and Monte Carlo simulation, such as thermodynamic integration (TI) [12] and free energy perturbation (FEP) [13]. These methods have been used to calculate hydration free energy of amino acids [14], nucleobases [15], and ligand/protein binding free energy [16,17,18,19,20,21,22,23,24,25,26,27,28]. They can treat water molecules either implicitly or explicitly. Thus, in the case of ligand/protein binding free energy calculation, they can be used to predict the thermodynamic behavior of a ligand in solvents. In compounds containing water molecules bridging between the protein and ligand, the calculation with explicit water molecules is efficient for calculating binding free energy. In the application of alchemical free energy calculation to log*P_ow_* calculation, DeBolt and Kollman successfully estimated the log*P_ow_* using FEP method with modified force field [29]. They were able to reproduce the structural and thermodynamic behaviors of liquid octanol. Using an implicit solvation model, Huang et al. reported a log*P_ow_* prediction method in which the 3D-RISM-KH theory was used to calculate the solvation free energy in water and in octanol [30]. To reproduce the experimental conditions, Bhatnagar et al. reported an adaptive biasing force method using explicit water and octanol solvents [31]. In another application, saturated octanol solvation was used for the calculation, taking the hydrate state of octanol in the experiment into account [32,33]. These methods were able to estimate log*P_ow_* values in agreement with the ones measured experimentally. The alchemical methods using all-atom MD simulation require time and computer resources, compared with atom- and fragment-based methods. However, these methods are able to predict the partition coefficient of several solvents, and observe the thermodynamic behavior of compounds in the solvents. Therefore, the free energy calculation using the alchemical method is expected to provide useful information on physical chemistry properties of the compounds.

In this paper, the log*P_ow_* values for relatively small compounds were predicted from the solvent free energies calculated by TI [34,35]. The calculation of the difference free energy, ∆*G*, was estimated by the Bennett’s acceptance ratio (BAR) method [36]. To analyze the contribution of the atomic charges of the compounds, three sets of atomic charges were examined: one in vacuum, one in octanol, and the other in water. Solvation free energies in water and in octanol are in good agreement with the ones measured experimentally. The correlation coefficient (*R*) of the solvation free energies indicated strong correlation between calculated and experimentally measured values. They did not differ significantly among the three sets of atomic charges. The log*P_ow_* values, however, showed slightly different behaviors. In fact, the *R* values of log*P_ow_* depended on the combination of the atomic charges calculated in the different solvents. These results reveal that the environment assumed in the calculation of the atomic charges is a very critical factor for the log*P_ow_* calculations. Therefore, our results provide useful information for improving the quality of the calculations. Finally, the log*P_ow_* values of 17 drugs calculated using the best combination of the atomic charges showed good agreement with the values measured experimentally.

## 2. Results and Discussion

### 2.1. Solvation Free Energy in Water

To calculate free energy in water, the three charge sets were used independently. In this paper, the notation of the parameter sets calculated with the atomic charges in the different solvents are simplified as *P_water_*{*x*}, where *x* is either *v*(vacuum), *o*(octanol), or *w*(water). It should be noted that, in this work, we did not change the atomic charges in water molecules, because the force field of water molecules was established by the many studies conducted on water molecules [37].

**Figure 1:**
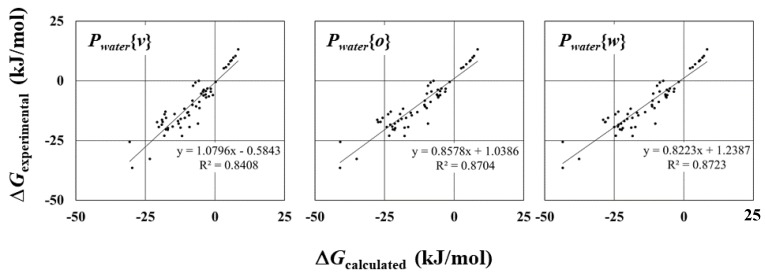
Scatter diagram of calculated and experimentally measured ∆*G* values in water solvent. *R*^2^ and regression line are shown.

Strong correlations between calculated and experimentally measured ∆*G_water_* values were observed (Figure 1). In particular, the free energy calculated using *P_water_*{*w*}, for which the coefficient of determination (*R*^2^) is 0.87, indicated good agreement with experimentally measured values. The free energy calculated using *P_water_*{*v*} also showed a strong correlation with the experimental data, even though *R*^2^ (=0.84) was the smallest in the three cases. These results confirm that the calculated atomic charges in the different environments do not necessarily influence the correlation between calculated and experimentally measured ∆*G_water_* values.

**Figure 2:**
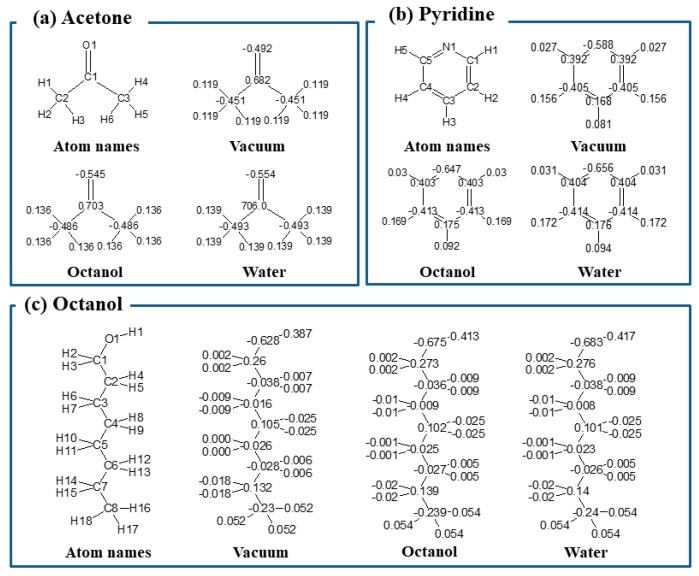
Calculated atomic charges of (**a**) Acetone, (**b**) Pyridine, and (**c**) Octanol in vacuum, octanol, and water.

On the other hand, the values of atomic charge were sensitive to the solvent in the quantum chemistry calculation (Figure 2). In the three solvents, somewhat large differences in atomic charge values can be found in the charged group containing heteroatoms, e.g., oxygen in acetone, nitrogen in pyridine, and oxygen in octanol. The calculated atomic charges in octanol and water were similar, and were slightly different from the ones in the vacuum state. This tendency can be understood from the graphs drawn for the atomic charges vs. dielectric constant values (Figure S1). The atomic charges dramatically increase or decrease at dielectric constants around 10, and become constant thereafter. These differences can affect the regression line in the scatter diagrams (Figure 1). The slope of the regression line for ∆*G_water_* values with *P_water_*(*w*) is close to that with *P_water_*(*o*), whereas that with *P_water_*(*v*) is slightly different from the others. However, the *R* values do not differ significantly among the three parameters. Furthermore, there were strong correlations among three.

∆*G_water_* values, for which the *R* values of *P_water_*(*v*)–*P_water_*(*o*), *P_water_*(*v*)–*P_water_*(*w*), and *P_water_*(*o*)–*P_water_*(*w*) pairs, were 0.9912, 0.9885, and 0.9996, respectively. In these cases, the order in which the compounds are arranged according to the ∆*G_water_*, is more or less the same among the three pairs. These analyses show that the atomic charges were affected by the solvent types assumed in the calculation, but these differences did not affect the correlation of the ∆*G_water_* values.

The RMSE and MAE values in the three parameter sets show opposite relations to the *R* values; i.e., the calculated data having strong correlation showed large errors (Table 1). The ∆*G_water_* of *P_water_*{*v*} had the smallest *R* and *R*^2^ values, but the errors, for which the RMSE and MAE values are 4.49 kJ/mol (=1.07 kcal/mol) and 3.65 (=0.87 kcal/mol), respectively, were the smallest in the three parameter sets. On the other hand, the calculation with *P_water_*{*w*} shows the largest *R* value, but the RMSE and MAE values are the largest. In these calculations, the difference of RMSE and MAE values, however, are 1.2 kJ/mol and 0.6 kJ/mol between *P_water_*{*v*} and *P_water_*{*w*}. These may not be significant differences among the three parameters. Therefore, for all the selections of atomic charges, the BAR method can estimate the ∆*G_water_* values accurately enough.

### 2.2. Solvation Free Energy in Octanol

In calculating the free energy in octanol, nine sets of the atomic charges were used: all combinations of the three sets of atomic charges of the compound (3 sets) and octanol (3 sets). The atomic charges of octanol molecule in octanol solvation, the octanol environment, should be considered. However, in this work, to understand the effect of the atomic charges in octanol on the log*P_ow_* calculation, we took into account the atomic charges in the octanol molecule for vacuum, octanol, and water state.

The compound and octanol parameter sets calculated with the atomic charges in octanol is defined as *P_octanol_*{*x*, *y*}, in which *x* and *y* (for compound and octanol, respectively) are either *v*(vacuum), *o*(octanol), or *w*(water).

**Figure 3:**
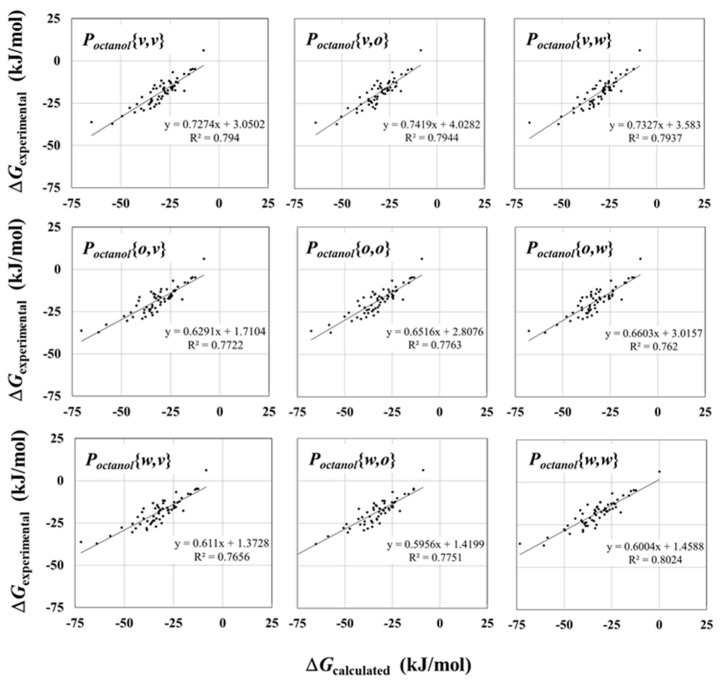
Scatter diagram of calculated and experimentally measured ∆*G* values in octanol solvent. *R*^2^ and regression line are shown.

Tendencies similar to those seen in the results obtained with the ∆*G_water_* calculation could be seen in the results obtained with the ∆*G_octanol_* calculation. It can be seen in Figure 3 that all the calculated ∆*G_octanol_* values showed strong correlations with the ones measured experimentally. The largest *R* value was 0.9 (*P_octanol_*{*v*, *o*} and *P_octanol_*{*v*, *w*}), though these values are slightly smaller than that in ∆*G_water_* (0.93). The calculated ∆*G_octanol_* values distributed toward the small range of the horizontal axis, thus, all the calculations may underestimate the ∆*G_octanol_* value. The tendency of the relationship between the *R*^2^ values and error values (RMSE and MAE) is similar to that seen in the ∆*G_water_* results; i.e., the larger the *R*^2^ values are, the larger the RMSE and MAE values are. The largest MAE is 13.26 kJ/mol (=3.16 kcal/mol) in the ∆*G_octanol_* with *P_octanol_*{*w*, *w*}. This value is a little bit larger than that in ∆*G_water_*. This result reveals that the calculation of ∆*G_octanol_* values accumulates more errors than that of ∆*G_water_*. However, these ∆*G_octanol_* values still showed strong correlation with the experimental data.

In Figure 3 and Table 2, the correlations between the calculated and experimentally measured ∆*G_octanol_* values are shown to be independent of the atomic charges. The values of *R* and *R*^2^ are close in all the ∆*G_octanol_* calculations. The *R*, *R*^2^, RMSE, and MAE values of the ∆*G_octanol_* with *P_octanol_*{*o*, *o*} are comparable with the other ones. This means that the atomic charges did not affect the correlation of ∆*G_octanol_* calculations. The ∆*G_octanol_* calculated with either *P_octanol_*{*v*, *o*} or *P_octanol_*{*v*, *w*} had the largest *R* and *R*^2^ values. Those values indicated a strong correlation with the experimental data. On the other hand, the ∆*G_octanol_* calculated with *P_octanol_*{*w*, *v*} had the smallest *R* and *R*^2^ values. However, these values still indicated strong correlation. The differences of *R* and *R*^2^ values between *P_octanol_*{*v*, *o*} (or *P_octanol_*{*v*, *w*}) and *P_octanol_*{*w*, *v*}, are 0.02 and 0.04, respectively. These are not necessarily significant differences. Therefore, it is possible to conclude that the calculated atomic charges in the different environments did not affect the *R* and *R*^2^ values in ∆*G_octanol_* calculations.

### 2.3. Effect of Water and Octanol Solvents on Free Energy

To investigate the effects of solvents on free energy calculation, the dielectric constant of water and octanol solvents were evaluated in the simulation. The dielectric constant was calculated as [38,39]

(1) $$\varepsilon =1+\frac{4\pi }{3k_{b}T\langle V\rangle }\left\{\langle M^{2}\rangle -{\langle M\rangle }^{2}\right\}$$

where *V*, *k_b_* and *T* are volume, Boltzmann constant, and temperature, respectively. *M* is the total dipole moment. To calculate the dielectric constant of water and octanol solvents, additional 5 ns MD simulations were performed to generate the products, after 10,000 steps optimization, 50 ps heat-up, and 2 ns equilibration. The dielectric constants were calculated for the trajectories of 5 ns MD simulations.

Overestimated dielectric constants of water solvents were obtained by simulation (Table 3). This value is very similar to that reported by Vega et al. [40]. In Figure 1, the largest slope of the regulations line is found in the calculation with *P_water_*(*v*), of which absolute atomic charge values was smallest in the three parameters. Therefore, the pair of the water solvent with which the dielectric constant was underestimated, and the compound with *P_water_*(*v*) provided the closest absolute values of ∆*G* in our calculation.

On the other hand, the dielectric constants of octanol solvent were also underestimated. All the values in the different charges were smaller than the experimentally measured ones. These values were slightly smaller than that in the papers reported by DeBolt and Kollman [29]. They obtained dielectric constants near 5.1 for the liquid octanol at 40 °C. The dielectric constant in octanol solvent gradually decreased by the atomic charges in the order of vacuum, octanol, and water, i.e., these values decreased in proportion to the absolute values of atomic charges. In Table 3, <*μ*> of octanol with atomic charge in water was larger than that of vacuum because of increasing absolute atomic charges. This means that, owing to increasing <*μ*>, the values of electrostatic interaction increased. On the other hand, the van der Waals interaction drops with increasing absolute atomic charges. Since the van der Waals parameters were the same among the different simulations, the electrostatic interaction is thought to dominate the simulation. However, in Figure 3, the slopes of the regulation line were dependent on the atomic charges of compounds, rather than those of octanol solvents. Moreover, the dielectric constant with atomic charges in water solvent further differed from the experimentally measured values than that within vacuum. In the simulation of liquid octanol, of which the dielectric constant of the octanol is relatively smaller than that of water solvent, a strong contribution of the electrostatic interaction leads to smaller dielectric constant values that appear as simulation errors. Therefore, in order to improve the calculated free energy values, the balance between electrostatic and van der Waals interactions may need to be investigated in further studies.

### 2.4. LogP_ow_ for Tested Compounds

The log*P_ow_* values were calculated using the Formula (7) (See Section 3). Here, the notation for the log*P_ow_* value calculated from ∆*G_water_* using *P_water_*{*x*}, and ∆*G_octanol_* using *P_octanol_*{*y*, *z*} was simplified to *P_logP_*{*x*, {*y*, *z*}}, where *x*, *y*, and *z* are either *v*(vacuum), *o*(octanol), or *w*(water).

**Figure 4:**
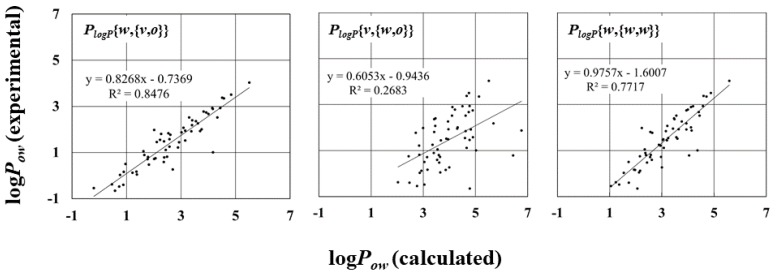
Scatter diagram of calculated and experimentally measured log*P_ow_* values. The diagrams with the largest and smallest *R*^2^ are shown, and the diagram of *P_logP_*{*w,* {*w, w*}} are shown for reference. *R*^2^ and regression line are shown.

The log*P_ow_* values were sensitive to the atomic charges calculated in the solvent. The correlation of the log*P_ow_* with the experimental data is strongly dependent on the pair of solvation free energies ∆*G_water_* and ∆*G_octanol_* (Figure 4 and Figure S2). For instance, in Figure 4, completely different correlations of log*P_ow_* values can be seen in the scatter diagrams with the largest and smallest *R*^2^, which are *P_logP_*{*w*, {*v*, *o*}} and *P_logP_*{*v*, {*w*, *o*}}, respectively. In the case of *P_logP_*{*w*, {*v*, *o*}}, the log*P_ow_* was calculated from ∆*G_water_* with *P_water_*{*w*}, and ∆*G_octanol_* with *P_octanol_*{*v*, *o*}. This combination is expected to result in log*P_ow_* values closely correlated with the experimental ones.

On the other hand, in the case of *P_logP_*{*v*, {*w*, *o*}}, the log*P_ow_* values showed very weak correlation with the experimental data. The calculated log*P_ow_* values were based on ∆*G_water_* with *P_water_*{*v*} and ∆*G_octanol_* with *P_octanol_*{*w*,*o*}. The free energies in water and octanol both showed strong correlations with the experimental data, and the RMSE and MAE values were not as significantly different from the others, as mentioned above. The slope of the regression line for ∆*G_water_* with *P_water_*{*v*}, however, was over 1, whereas that of the regression line for ∆*G_octanol_* with *P_octanol_*{*w*,*o*} was under 1. These differences raised the large RMSE and MAE values slightly (Table 4). As a result, the log*P_ow_* values showed very weak correlation. These results reveal that the combination of the ∆*G_water_* and ∆*G_octanol_* is very important for the correct log*P_ow_*. That is, the selection of the solvent in the calculation of the atomic charges is very important for the log*P_ow_* calculation. Thus, the inappropriate selection of the solvents for the calculation of atomic charges results in the weak correlation between the log*P_ow_* and the experimental data. This tendency can be observed in the log*P_ow_* with *P_logP_*{*w*,{*w*, *w*}}. The ∆*G_water_* with *P_water_*{*w*}, and ∆*G_octanol_* with *P_octanol_*{*w*, *w*}, showed the strongest correlation in our test data. The values, however, showed moderate correlation (*R* = 0.77) not stronger than that of the ∆*G_water_* with *P_logP_*{*w*, {*v*, *o*}}.

**Figure 5:**
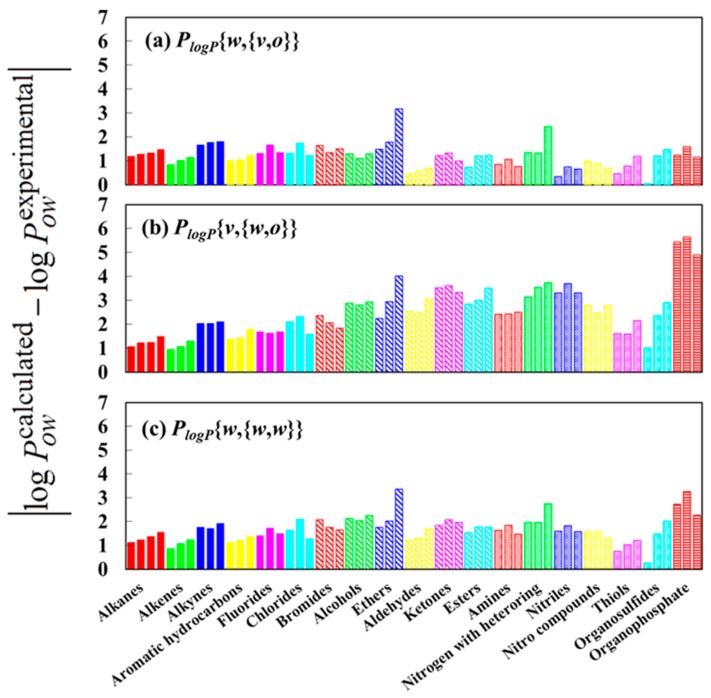
Error of log*P_ow_* with (a) *P_logP_{w, {v, o}}*, (b) *P_logP_{v, {w, o}}*, and (c) *P_logP_{w, {w, w}}* for the experimental values. Here, the left-to-right order of the compounds in each group is the same as the top-down order in Table S1.

The chemical group’s effect on the log*P_ow_* values of a compound is shown in Figure 5. In the log*P_ow_* calculation with *P_logP_*{*w*, {*v*, *o*}}, the errors of each compound were ~2.0 in all groups, except the ethers (for ethyl phenyl ether, the error was 3.16). The calculated ∆*G_water_* value of ethyl phenyl ether was an overestimate. The calculated and the experimentally measured ∆*G_water_* values were −10.22 kJ/mol and −17.92 kJ/mol, respectively. On the other hand, the calculated ∆*G_octanol_* was an underestimate. The calculated and experimentally measured ∆*G_octanol_* values were −34.91 kJ/mol and −23.65 kJ/mol, respectively. These differences caused large errors in log*P_ow_* values. Similar errors can be found in the log*P_ow_* values calculated with *P_logP_*{*w*, {*w*, *w*}}. The profile of histograms for *P_logP_*{*w*, {*w*, *w*}} is similar to that of histograms for *P_logP_*{*w*, {*v*, *o*}}. The error of ethyl phenyl ether stands out from the other compounds. The error of each compound was around 2.0, but these errors are slightly larger than that of the values calculated with *P_logP_*{*w*, {*v*, *o*}}. Therefore, the *R* values for *P_logP_*{*w*, {*w*, *w*}} were slightly smaller than those for *P_logP_*{*w*, {*v*, *o*}}.

On the other hand, most of the log*P_ow_* values calculated with *P_logP_*{*v*, {*w*, *o*}} were larger than those calculated with the other parameters. Those of the compounds in the phosphate group, which has a strong polarity at the phosphoric acid moiety, showed particularly large errors. The selected environments in the charge calculation was inconsistent, i.e., the vacuum state was selected in ∆*G_water_* calculation and water environment was selected in ∆*G_octanol_* calculations. These inadequate combinations induce large errors in the log*P_ow_* calculation. A similar tendency can be found in the other groups with polar moieties, such as ether and ketone groups. The selection of the environment of the calculated charges, therefore, is important in log*P_ow_* calculation.

To analyze the contributions of the atomic charges in different environments, the *R* and *R*^2^ values of log*P_ow_* in Table 4 were compared. In the calculated ∆*G_water_*, the *R* and *R*^2^ values become small in the order of *P_water_*{*v*}, *P_water_*{*o*}, and *P_water_*{*w*} (Figure S3a). In terms of the atomic charges of the compounds in ∆*G_octanol_* calculations, the *R* and *R*^2^ values of log*P_ow_* become small in the order of water, octanol, and vacuum environments (Figure S3b). However, the atomic charges of the octanol in octanol solvent seem to affect the log*P_ow_* values very slightly (Figure S3c). To briefly summarize these results, the atomic charges of the compound in the ∆*G_water_* and ∆*G_octanol_* calculations are better in water and vacuum environments, respectively. In free energy, the atomic charges of the octanol molecule in octanol solvent should be reasonable, nonetheless, they do not influence the log*P_ow_* value much. It should be noted that this tendency will be independent of the functional and basis sets in the calculation of atomic charges, because the RMSE shows the same behavior (Figure S4), and the correlation efficient values of our procedure are very large with the other procedures using the different functionals and basis sets in five compounds (Table S4).

### 2.5. LogP_ow_ for 17 Compounds

The best combination of the parameters of the additional 17 compounds (*P_logP_*{*w*, {*v*, *w*}}) was different from those of the small compounds (*P_logP_*{*w*, {*v*, *o*}}) (Table S5). However, the *R* and *R*^2^ values of *P_logP_*{*w*, {*v*, *v*}}, *P_logP_*{*w*, {*v*, *o*}}, and *P_logP_*{*w*, {*v*, *w*}} were very close to each other. Therefore, we conclude that choosing one of *P_logP_*{*w*, {*v*, *}} (* = *v*, *o*, and *w*) parameter set will provide *R* and *R*^2^ values close to the experimental ones.

**Figure 6:**
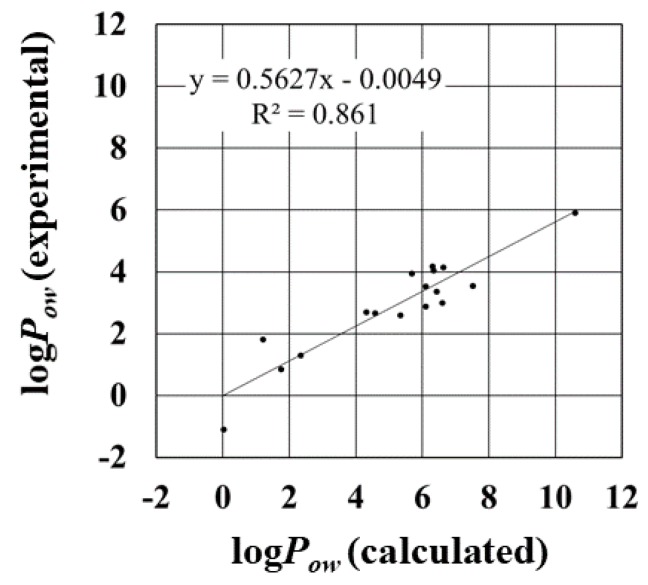
Scatter diagram of calculated and experimentally measured log*P_ow_* values for 17 compounds.

The calculated log*P_ow_* values of the additional 17 compounds with the best combination of the atomic charges in water and octanol (*P_logP_*{*w*, {*v*, *w*}}) showed a good agreement with the experimental data (Figure 6). The *R* and *R*^2^ values were 0.93 and 0.86, respectively. These values were similar to those obtained by the other methods based on the compounds’ atoms and fragments [44], though our method needs more computational time than the other methods. The RMSE and MAE were 2.58 and 2.26, respectively, which were slightly larger than those of the small compounds. These errors affect the slope of the regression line, which is somewhat smaller than that of the line obtained for the small compounds when using *P_logP_*{*w*, {*v*, *o*}} (Figure 4). These results indicate that the order of the compounds sorted by the log*P_ow_* values, is very similar to the order of the compounds sorted by the log*P_ow_* values measured experimentally. This means that the alchemical free energy calculation method with *P_logP_*{*w*, {*v*, *w*}} will be effective to predict the lipophilicity of compounds, and will be a useful tool for drug design.

**Figure 7:**
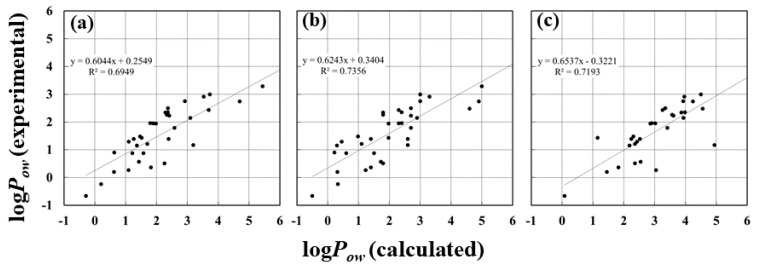
Scatter diagrams of calculated using (**a**) GAFF, (**b**) GAFF-DC, and (**c**) *P_logP_*{*w*, {*v*, *o*}}, and experimentally measured log*P_ow_* values for 32 compounds*.* The values calculated by GAFF and GAFF-DC, and the experimentally measured values were taken from the reference by Bannan et al. [45].

The log*P_ow_* values of the other additional 32 compounds were calculated for comparing our results with the previous results reported by Bannan et al. [45]. The log*P_ow_* values show strong correlation with the experimentally measured values, of which the *R* and *R*^2^ values were 0.85 and 0.72, respectively (Figure 7). These values are very close to those calculated using GAFF-DC that were reported by Bannan et al., of which *R* and *R*^2^ values are 0.85 and 0.74, respectively. These results reveal that the consideration of the atomic charge is important for improving the log*P_ow_* values.

## 3. Materials and Methods

### 3.1. Setting Parameters of Compounds

The general amber force field (GAFF) [46] was used for bonding parameters (bond length, bond angle, torsion angle, and improper torsion angle) and for nonbonding van der Waals parameters (radius and well-depths) of the compounds and 1-octanol. Atomic charges were calculated by using Gaussian09 software [47] at the B3LYP/6-31G(d) level. The polarizable continuum model (PCM) [48] was used to consider the solvent effect on the calculation of atomic charges. Antechamber software was used for restrained electrostatic potential (RESP) charge-fitting [49,50,51] from the results of quantum chemical calculation. In the preparations for the simulations, biomolecule’s atomic charges are calculated once in one solvent, i.e., in water solvent, in protein, or in vacuum state. When calculating a compound’s free energy in octanol, however, better results can be obtained using the atomic charges calculated for the compound in octanol. Therefore, in this study, the atomic charges of the compounds and 1-octanol were calculated for three states: in vacuum, in 1-octanol, and in water. In the calculation of the free energy in water, the three charge sets were used independently. To calculate the free energy in octanol, nine sets of the atomic charges were used: all combinations of the three sets of atomic charges of the compound (3 sets) and octanol (3 sets). The compound and octanol parameter sets calculated with the atomic charges in octanol is defined as *P_octanol_*{*x*, *y*}, in which *x* and *y* (for compound and octanol, respectively) are either *v*(vacuum), *o*(octanol), or *w*(water). The log*P_ow_* values based on these free energies were calculated. The dependency of the calculated atomic charges in the different environments on the free energy and log*P_ow_* values were examined.

### 3.2. Initial Models of Compounds in Solvent Box

We constructed the model systems as follows. All the compounds were hydrated using a TIP3P [37] water box consisting of 723 water molecules, and all the compounds were placed into an octanol box consisting of 100 1-octanol molecules.

All the systems were minimized by 10,000 steps of the steepest-descent method, first with constraint on the positions of the heavy atoms, and then without constraint. After the minimization, 7 ns MD simulation for the compound was performed with Gromacs 4.5.3 software suite in a periodic boundary condition, by using particle-mesh Ewald (PME) calculations [52,53] for coulombic interactions. The temperature was set at 298.15 K by using a stochastic dynamics algorithm, and pressure was maintained at 1 bar by scaling the box size in accordance with the Berendsen algorithm [54]. The frictional coefficient γ and time step were 5 ps^−1^ and 2.0 fs. Snapshot structures were extracted from equilibration runs as initial structures for successive annihilation MD simulations. The snapshot after 2 ns of MD simulation was used as the initial structure for the free energy calculation.

### 3.3. Free Energy Calculation with BAR Method

Annihilation with the BAR method is widely used in the calculation of ligand/protein binding free energy [25,26,27,28], and is briefly explained here. We consider the changes in free energy for transformation from vacuum to solvent using the BAR method, based on the following formulas:

(2) $$\Delta G=\Delta G_{solvent}-\Delta G_{gas}$$

(3) $$S_{gas}\overset{\;\Delta G_{solvent}\;}{\to }S_{solvent}$$

(4) $$S_{0}\overset{\;\Delta G_{gas}\;}{\to }S_{gas}$$

where Δ*G_solvent_* represents the free energy change from the compound in gas (*S_gas_*) to the compound in solvent (*S_solvent_*) in Formula (3), and Δ*G_gas_* represents the free energy change from the compound in vacuum (*S*_0_) to the compound in gas (*S_gas_*) in Formula (4). In Formula (3), to calculate the interaction by considering the reverse processes, the first annihilation is performed for the compound from solvent to gas. And in Formula (4), the second annihilation is performed to remove the internal energy in the compounds. In these processes, the free energy is estimated by summing the free energy differences between neighboring partially annihilated states (here states are denoted as windows). The free-energy difference between the neighboring *i*-th and (*i* + 1)-th windows is calculated by using the BAR method [55]. In the BAR method, for *U_i_* representing the potential energy of the *i*-th window, nonequilibrium work *W* (=*U_i_*_+1_–*U_i_*) was sampled in the MD trajectory. According to the maximum likelihood argument of BAR, the free-energy estimator for two equal-length simulations can be written as

(5) $$<{\left[1+\exp\left\{\left(W-\Delta G_{i}\right)/RT\right\}\right]}^{-1}>{}_{i}=<{\left[1+\exp\left\{-\left(W-\Delta G_{i}\right)/RT\right\}\right]}^{-1}>{}_{i+1}$$

where Δ*G_i_* is the annihilation free-energy change from the *i*-th window to the (*i* + 1)-th window, and where a pair of angle brackets indicates an ensemble average over a window. The free energy difference is then defined as follows:

(6) $$\Delta G=\sum_{i}\Delta G_{i}$$

In the free energy calculation using the BAR method, the annihilating simulations with the soft core potential [56,57] were independently performed for the sets of *λ* points composing *λ*_C_ and *λ*_LJ_ for Coulomb and van der Waals interactions, respectively. With *λ*_LJ_ = 0.0, the *λ*_C_ was increased in steps of 0.05 from 0 to 1, and with *λ*_C_ = 1, the *λ*_LJ_ was increased in steps of 0.05 from 0 to 1. Totally, 41 *λ*-points were considered in this paper.

For each window, 1 ns runs for data collection were performed for water and octanol solvent after 1 ns MD simulation for equilibration. Nonequilibrium work between neighboring windows was measured every 2 ps. Other conditions for each simulation were the same as those for the simulation generating initial structure, as mentioned above. To reduce artifacts due to the initial structure selection and to improve the statistical reliability, three independent sets of annihilation simulations were performed.

### 3.4. Calculation of LogP_ow_

The log*P_ow_* values were calculated using the following formula:

(7) $$\begin{array}{ll} \log P_{ow} & =-\frac{\Delta G_{transfer}^{octanol\to water}}{2.303\;RT}\\ & =-\frac{\Delta G_{octanol}-\Delta G_{water}}{2.303\;RT} \end{array}$$

where *R* and *T* are the gas constant and temperature, respectively, and Δ*G_octanol_* and Δ*G_water_* are the free energy differences (calculated using the BAR method as described) between the compound in solvent and the compound in vacuum. Here, the notation for the log*P_ow_* value calculated from Δ*G_water_* using *P_water_*{*x*}, and Δ*G_octanol_* using *P_octanol_*{*y*, *z*}, was simplified to *P_logP_*{*x*, {*y*, *z*}}, where *x*, *y*, and *z* are either *v*(vacuum), *o*(octanol) or *w*(water). Equation (6) indicates that we should consider the effects of free energy differences caused by the perturbation of the atomic charge, by the change of the environments, and by the different atomic charges. In this work, these free energy differences were neglected, because they cannot be accurately reproduced using currently available methods.

### 3.5. Test Compounds

The 58 relatively small sized compounds examined to measure the log*P_ow_* are listed in Table S1, along with their experimentally measured free energies extracted from a paper by Wang et al. [58]. In each compound, log*P_ow_* values were calculated by the combination of the free energies of the water and octanol solvents, i.e., three *P_water_*{*x*} and nine *P_octanol_*{*x*, *y*} parameters. Finally, 27 log*P_ow_* values were calculated in total.

The log*P_ow_* values of an additional 17 compounds were calculated using the same combination of the atomic charges in water and octanol for free energy calculations, as mentioned above. These compounds (Table S2) were used as drugs in the work reported by Daina et al. [44]. The calculation conditions used were the same as those used for the other 58 compounds.

As additional tests, the log*P_ow_* values of 32 compounds tested in the previous work reported by Bannan et al. [45], were calculated using the best combination of the atomic charge calculations. In this case, the *P_water_*{*w*} and *P_octanol_*{*v*, *o*} showed the best correlation, and were used in additional calculations. All the calculations were performed with the same conditions as the previous work by Bannan et al. [45], except for the set of *λ* points, for which the values of this work were used, as mentioned above.

## 4. Conclusions

A log*P_ow_* calculation method using alchemical free energy calculation with the BAR method was proposed, and the results showed good agreement with the values measured experimentally. The free energies in water and octanol both showed strong correlations with the experimental data. However, the correlation of log*P_ow_* values was sensitive to the combination of the Δ*G_water_* and Δ*G_octanol_* values. The weak correlation was due to the different slopes of regression line between Δ*G_water_* and Δ*G_octanol_*. Finally, we found that the calculation with *P_logP_*{*w*, {*v*, *o*}} provides the best correlations between the calculated log*P_ow_* values and experimentally measured values.

We calculated the log*P_ow_* values for the drugs using the best parameter set in our trials, and obtained values closely correlated with the ones obtained experimentally. The *R* values of log*P_ow_* values were better than those obtained in previous studies. These results show that the solvent assumed in the charge calculation greatly influences the log*P_ow_* values. The calculation of the atomic charges was time consuming, but dramatically increased the accuracy of the log*P_ow_* values. We expect these accurate results to be useful in the field of drug discovery, and to accelerate drug design processes.

## Figures and Tables

**Table 1:** Summary of free energy in water calculations.

Parameter	*R*	*R*^2^	RMSE ^a^ (kJ/mol)	MAE ^b^ (kJ/mol)
*P_water_*{*v*}	0.92	0.84	4.47	3.63
*P_water_*{*o*}	0.93	0.87	5.05	3.74
*P_water_*{*w*}	0.93	0.87	5.69	4.28

^a^ Root mean square error; ^b^ Mean average error.

**Table 2:** Summary of free energy in octanol calculations.

Parameter	*R*	*R*^2^	RMSE ^a^ (kJ/mol)	MAE ^b^ (kJ/mol)
*P_octanol_*{*v*, *v*}	0.89	0.80	11.18	10.36
*P_octanol_*{*v*, *o*}	0.90	0.81	11.23	10.49
*P_octanol_*{*v*, *w*}	0.90	0.81	11.26	10.56
*P_octanol_*{*o*, *v*}	0.88	0.78	13.59	12.53
*P_octanol_*{*o*, *o*}	0.89	0.78	13.70	12.77
*P_octanol_*{*o*, *w*}	0.89	0.79	13.65	12.72
*P_octanol_*{*w*, *v*}	0.88	0.77	14.18	13.05
*P_octanol_*{*w*, *o*}	0.89	0.79	14.15	13.18
*P_octanol_*{*w*, *w*}	0.89	0.79	14.23	13.26

^a^ Root mean square error; ^b^ Mean average error.

**Table 3:** Properties of solvents in simulation.

Solvents	Charge	ε		<*μ*> (Debye)	Flu ^c^ (Debye^2^)	<V> (Å^3^)	vdW (kJ/mol)	Elec. (kJ/mol)
		Exp.	Sim.					
Water		78.49 (25 °C) ^a^	92.70	2.35	20,914.2	22,290.7	14,371.9	−33,641.5
Octanol	Vacuum	9.34 (32.1 °C) ^b^	4.21	2.08	836.1	26,547.7	−3860.9	−2778.5
	Octanol		3.63	2.24	678.0	26,320.5	−3768.3	−3687.8
	Water		3.42	2.27	625.2	26,347.2	−3714.9	−3879.3

^a^ Uematsu and Frank [41]; ^b^ Smyth and Stoops [42,43]; ^c^ Flu = <*M***^2^**> − <*M*>^2^.

**Table 4:** Summary of log*P_ow_* calculations.

Parameters	*R*	*R*^2^	RMSE ^1^ (kJ/mol)	MAE ^2^ (kJ/mol)
*P_logP_*{*v*, {*v*, *v*}}	0.77	0.59	2.16	2.04
*P_logP_*{*v*, {*v*, *o*}}	0.80	0.64	2.17	2.07
*P_logP_*{*v*, {*v*, *w*}}	0.82	0.68	2.17	2.07
*P_logP_*{*v*, {*o*, *v*}}	0.55	0.31	2.61	2.42
*P_logP_*{*v*, {*o*, *o*}}	0.60	0.36	2.63	2.46
*P_logP_*{*v*, {*o*, *w*}}	0.62	0.38	2.62	2.45
*P_logP_*{*v*, {*w*, *v*}}	0.49	0.24	2.72	2.51
*P_logP_*{*v*, {*w*, *o*}}	0.54	0.29	2.72	2.53
*P_logP_*{*v*, {*w*, *w*}}	0.54	0.29	2.74	2.55
*P_logP_*{*o*, {*v*, *v*}}	0.92	0.84	1.40	1.31
*P_logP_*{*o*, {*v*, *o*}}	0.92	0.85	1.42	1.34
*P_logP_*{*o*, {*v*, *w*}}	0.92	0.85	1.43	1.35
*P_logP_*{*o*, {*o*, *v*}}	0.85	0.73	1.79	1.69
*P_logP_*{*o*, {*o*, *o*}}	0.87	0.76	1.82	1.73
*P_logP_*{*o*, {*o*, *w*}}	0.88	0.77	1.81	1.72
*P_logP_*{*o*, {*w*, *v*}}	0.82	0.67	1.90	1.78
*P_logP_*{*o*, {*w*, *o*}}	0.84	0.71	1.90	1.81
*P_logP_*{*o*, {*w*, *w*}}	0.84	0.71	1.92	1.82
*P_logP_*{*w*, {*v*, *v*}}	0.92	0.85	1.26	1.17
*P_logP_*{*w*, {*v*, *o*}}	0.92	0.86	1.29	1.20
*P_logP_*{*w*, {*v*, *w*}}	0.92	0.85	1.31	1.21
*P_logP_*{*w*, {*o*, *v*}}	0.88	0.78	1.63	1.55
*P_logP_*{*w*, {*o*, *o*}}	0.90	0.80	1.66	1.59
*P_logP_*{*w*, {*o*, *w*}}	0.90	0.81	1.66	1.58
*P_logP_*{*w*, {*w*, *v*}}	0.86	0.75	1.73	1.64
*P_logP_*{*w*, {*w*, *o*}}	0.88	0.77	1.75	1.66
*P_logP_*{*w*, {*w*, *w*}}	0.88	0.77	1.76	1.68

^1^ Root mean square error; ^2^ Mean average error.

## Supplementary Materials

The following are available online, Table S1: Test compounds, free energy values, and log*P_ow_* values, Table S2: Additional test set, Table S3: Additional test set for comparing with our results. Table S4: Correlation coefficient of log*P_ow_* values using between B3LYP/6-31G* and the different methods and basis sets, Table S5: Summary of log*P_ow_* calculations for 17 compounds. Figure S1: Atomic charges vs. dielectric constant values in quantum chemistry calculation. Figure S2: Scatter diagram of calculated and experimentally measured log*P_ow_* values, and Figure S3: Bar graph of *R^2^* values for log*P_ow_*. Figure S4: RMSE of log*P_ow_* for five compounds (dimethyl sulfide, ethyl phenyl ether, naphthalene, 1-nitropropane, and tribromomethane) calculated using different methods and basis sets.

## References

[1] Hansch C., Fujita T. (1964). *p*-*σ*-π analysis. A method for the correlation of biological activity and chemical structure. J. Am. Chem. Soc..

[2] Leo A., Hansch C., Elkins D. (1971). Partition coefficients and their uses. Chem. Rev..

[3] Sangster J. (1989). Octanol–water partition coefficients of simple organic compounds. J. Phys. Chem. Ref. Data.

[4] Fujita T., Iwasa J., Hansch C. (1964). A new substituent constant, π, derived from partition coefficients. J. Am. Chem. Soc..

[5] Devoe H., Miller M.M., Wasik S.P. (1981). Generator columns and high pressure liquid chromatography for determining aqueous solubilities and octanol–water partition coefficients of hydrophobic substances. J. Res. Nat. Bur. Stand..

[6] Opperhuizen A., Serne P., Van der Steen J.M.D. (1988). Thermodynamics of fish/water and octan-1-ol/water partitioning of some chlorinated benzenes. Environ. Sci. Technol..

[7] Cheng T., Zhao Y., Li X., Lin F., Xu Y., Zhang X., Li Y., Wang R., Lai L. (2007). Computation of octanol–water partition coefficients by guiding an additive model with knowledge. J. Chem. Inf. Model..

[8] Meylan W.M., Howard P.H. (2000). Estimating log P with atom/fragments and water solubility with log P. Perspect. Drug Discov. Des..

[9] Lipinski C.A. (2000). Drug-like properties and the causes of poor solubility and poor permeability. J. Pharmacol. Toxicol. Methods.

[10] Bolton E.E., Wang Y., Thiessen P.A., Bryant S.H., Ralph A.W., David C.S. (2008). Chapter 12—Pubchem: Integrated platform of small molecules and biological activities. Annual Reports in Computational Chemistry.

[11] Irwin J.J., Sterling T., Mysinger M.M., Bolstad E.S., Coleman R.G. (2012). Zinc: A free tool to discover chemistry for biology. J. Chem. Inf. Model..

[12] Kirkwood J.G. (1935). Statistical mechanics of fluid mixtures. J. Chem. Phys..

[13] Zwanzig R.W. (1954). High-temperature equation of state by a perturbation method. I. Nonpolar gases. J. Chem. Phys..

[14] Shirts M.R., Pitera J.W., Swope W.C., Pande V.S. (2003). Extremely precise free energy calculations of amino acid side chain analogs: Comparison of common molecular mechanics force fields for proteins. J. Chem. Phys..

[15] Wolf M.G., Groenhof G. (2012). Evaluating nonpolarizable nucleic acid force fields: A systematic comparison of the nucleobases hydration free energies and chloroform-to-water partition coefficients. J. Comput. Chem..

[16] Chodera J.D., Mobley D.L., Shirts M.R., Dixon R.W., Branson K., Pande V.S. (2011). Alchemical free energy methods for drug discovery: Progress and challenges. Curr. Opin. Struct. Biol..

[17] Steinbrecher T., Labahn A. (2010). Towards accurate free energy calculations in ligand protein-binding studies. Curr. Med. Chem..

[18] Gao J., Kuczera K., Tidor B., Karplus M. (1989). Hidden thermodynamics of mutant proteins: A molecular dynamics analysis. Science.

[19] Ha S., Gao J., Tidor B., Brady J.W., Karplus M. (1991). Solvent effect on the anomeric equilibrium in D-glucose: A free energy simulation analysis. J. Am. Chem. Soc..

[20] Jorgensen W.L., Ravimohan C. (1985). Monte carlo simulation of differences in free energies of hydration. J. Chem. Phys..

[21] Kollman P. (1993). Free energy calculations: Applications to chemical and biochemical phenomena. Chem. Rev..

[22] Bash P., Singh U., Langridge R., Kollman P. (1987). Free energy calculations by computer simulation. Science.

[23] Wong C.F., McCammon J.A. (1986). Dynamics and design of enzymes and inhibitors. J. Am. Chem. Soc..

[24] Merz K.M., Kollman P.A. (1989). Free energy perturbation simulations of the inhibition of thermolysin: Prediction of the free energy of binding of a new inhibitor. J. Am. Chem. Soc..

[25] Kitamura K., Tamura Y., Ueki T., Ogata K., Noda S., Himeno R., Chuman H. (2014). Binding free-energy calculation is a powerful tool for drug optimization: Calculation and measurement of binding free energy for 7-azaindole derivatives to glycogen synthase kinase-3β. J. Chem. Inf. Model..

[26] Okada O., Yamashita H., Takedomi K., Ono S., Sunada S., Kubodera H. (2013). Prediction of the binding affinity of compounds with diverse scaffolds by MP-CAFEE. Biophys. Chem..

[27] Fujitani H., Tanida Y., Ito M., Jayachandran G., Snow C.D., Shirts M.R., Sorin E.J., Pande V.S. (2005). Direct calculation of the binding free energies of fkbp ligands. J. Chem. Phys..

[28] Fujitani H., Tanida Y., Matsuura A. (2009). Massively parallel computation of absolute binding free energy with well-equilibrated states. Phys. Rev. E.

[29] DeBolt S.E., Kollman P.A. (1995). Investigation of structure, dynamics, and solvation in 1-octanol and its water-saturated solution: Molecular dynamics and free-energy perturbation studies. J. Am. Chem. Soc..

[30] Huang W., Blinov N., Kovalenko A. (2015). Octanol–water partition coefficient from 3D-RISM-KH molecular theory of solvation with partial molar volume correction. J. Phys. Chem. B.

[31] Bhatnagar N., Kamath G., Chelst I., Potoff J.J. (2012). Direct calculation of 1-octanol–water partition coefficients from adaptive biasing force molecular dynamics simulations. J. Chem. Phys..

[32] Hansen N., Hünenberger P.H., van Gunsteren W.F. (2013). Efficient combination of environment change and alchemical perturbation within the enveloping distribution sampling (eds) scheme: Twin-system eds and application to the determination of octanol–water partition coefficients. J. Chem. Theory Comput..

[33] Chen B., Siepmann J.I. (2000). Partitioning of alkane and alcohol solutes between water and (dry or wet) 1-octanol. J. Am. Chem. Soc..

[34] Pranata J., Jorgensen L.W. (1991). Monte carlo simulations yield absolute free energies of binding for guanine—Cytosine and adenine—Uracil base pairs in chloroform. Tetrahedron.

[35] Jorgensen W.L., Buckner J.K., Boudon S., Tirado-Rives J. (1988). Efficient computation of absolute free energies of binding by computer simulations. Application to the methane dimer in water. J. Chem. Phys..

[36] Bennett C.H. (1976). Efficient estimation of free energy differences from monte carlo data. J. Comput. Phys..

[37] Jorgensen W.L., Chandrasekhar J., Madura J.D., Impey R.W., Klein M.L. (1983). Comparison of simple potential functions for simulating liquid water. J. Chem. Phys..

[38] De Leeuw S.W., Perram J.W., Smith E.R. (1980). Simulation of electrostatic systems in periodic boundary conditions. I. Lattice sums and dielectric constants. Proc. R. Soc. A. Math. Phys. Sci..

[39] Zhang C., Hutter J., Sprik M. (2016). Computing the kirkwood G-factor by combining constant maxwell electric field and electric displacement simulations: Application to the dielectric constant of liquid water. J. Phys. Chem. Lett..

[40] Vega C., Abascal J.L.F. (2011). Simulating water with rigid non-polarizable models: A general perspective. Phys. Chem. Chem. Phys..

[41] Uematsu M., Frank E.U. (1980). Static dielectric constant of water and steam. J. Phys. Chem. Ref. Data.

[42] Smyth C.P., Stoops W.N. (1929). The dielectric polarization of liquids. Vi. Ethyl iodide, ethanol, normal-butanol and normal-octanol. J. Am. Chem. Soc..

[43] Smyth C.P., Stoops W.N. (1929). The dielectric polarization of liquids. Vii. Isomeric octyl alcohols and molecular orientation. J. Am. Chem. Soc..

[44] Daina A., Michielin O., Zoete V. (2014). iLOGP: A simple, robust, and efficient description of *n*-octanol/water partition coefficient for drug design using the GB/SA approach. J. Chem. Inf. Model..

[45] Bannan C.C., Calabró G., Kyu D.Y., Mobley D.L. (2016). Calculating partition coefficients of small molecules in octanol/water and cyclohexane/water. J. Chem. Theory Comput..

[46] Wang J., Wolf R.M., Caldwell J.W., Kollman P.A., Case D.A. (2004). Development and testing of a general amber force field. J. Comput. Chem..

[47] Frisch M.J., Trucks G.W., Schlegel H.B., Scuseria G.E., Robb M.A., Cheeseman J.R., Scalmani G., Barone V., Mennucci B., Petersson G.A. (2009). Gaussian 09.

[48] Tomasi J., Mennucci B., Cammi R. (2005). Quantum mechanical continuum solvation models. Chem. Rev..

[49] Bayly C.I., Cieplak P., Cornell W., Kollman P.A. (1993). A well-behaved electrostatic potential based method using charge restraints for deriving atomic charges: The RESP model. J. Phys. Chem..

[50] Cornell W.D., Cieplak P., Bayly C.I., Kollmann P.A. (1993). Application of RESP charges to calculate conformational energies, hydrogen bond energies, and free energies of solvation. J. Am. Chem. Soc..

[51] Cieplak P., Cornell W.D., Bayly C., Kollman P.A. (1995). Application of the multimolecule and multiconformational RESP methodology to biopolymers: Charge derivation for DNA, RNA, and proteins. J. Comput. Chem..

[52] Darden T., York D., Pedersen L. (1993). Particle mesh ewald: An *N*·log(*N*) method for Ewald sums in large systems. J. Chem. Phys..

[53] Essmann U., Perera L., Berkowitz M.L., Darden T., Lee H., Pedersen L.G. (1995). A smooth particle mesh Ewald method. J. Chem. Phys..

[54] Berendsen H.J.C., Postma J.P.M., Van Gunsteren W.F., Dinola A., Haak J.R. (1984). Molecular dynamics with coupling to an external bath. J. Chem. Phys..

[55] Shirts M.R., Bair E., Hooker G., Pande V.S. (2003). Equilibrium free energies from nonequilibrium measurements using maximum-likelihood methods. Phys. Rev. Lett..

[56] Beutler T.C., Mark A.E., van Schaik R.C., Gerber P.R., van Gunsteren W.F. (1994). Avoiding singularities and numerical instabilities in free energy calculations based on molecular simulations. Chem. Phys. Lett..

[57] Zacharias M., Straatsma T.P., McCammon J.A. (1994). Separation-shifted scaling, a new scaling method for lennard-jones interactions in thermodynamic integration. J. Chem. Phys..

[58] Wang J., Wang W., Huo S., Lee M., Kollman P.A. (2001). Solvation model based on weighted solvent accessible surface area. J. Phys. Chem. B.

