# Supplementary material for: Effect of Atomic Charges on Octanol–Water Partition Coefficient Using Alchemical Free Energy Calculation
Source: Molecules. 2018 Feb 15;23(2):425. doi: 10.3390/molecules23020425 (PMC6017306; doi:10.3390/molecules23020425)
Supplement: Supplementary file 1 [file molecules-23-00425-s001.pdf]

Supplementary Materials

Effect of Atomic Charges on Octanol-Water Partition Coefficient Using Alchemical Free Energy Calculation

Koji Ogata,* Makoto Hatakeyama, and Shinichiro Nakamura

RIKEN Innovation Center, Nakamura Laboratory, 2-1 Hirosawa, Wako, Saitama 351-0198, Japan; ogatak@riken.jp

* Correspondence: ogatak@riken.jp; Tel.: +81-(0)48 467-9477

Table S1. Test compounds, free energy values, and $\log P_{ow}$ values[1]

Group	Compound	ΔG_{water} (kJ/mol)	$\Delta G_{octanol}$ (kJ/mol)	$\log P_{ow}$
Alkanes	propane	8.20	−5.27	2.36
	n-butane	8.71	−7.79	2.89
	n-pentane	9.75	−10.26	3.50
	n-hexane	10.42	−12.60	4.03
Alkenes	propylene	5.32	−4.77	1.77
	1–butene	5.78	−7.91	2.40
	1-hexene	6.95	−12.31	3.37
Alkynes	propyne	−2.01	−6.66	0.81
	1-pentyne	−0.67	−11.68	1.93
	1-hexyne	0.04	−14.36	2.52
Aromatic hydrocarbons	benzene	−3.73	−15.57	2.08
	toluene	−3.18	−19.05	2.78
	naphthalene	−10.09	−29.18	3.34
Fluorides	1,1-difluoroethane	−0.46	−4.73	0.75
	tetrafluoromethane	13.23	6.28	1.22
	fluorobenzene	−3.27	−16.20	2.27
Chlorides	dichloromethane	−5.69	−12.85	1.25
	trichloromethane	−4.48	−15.95	2.01
	chlorobenzene	−4.23	−20.93	2.93
Bromides	dibromomethane	−8.83	−17.50	1.52
	tribromomethane	−8.29	−23.53	2.67
	bromobenzene	−6.11	−22.86	2.93
Alcohols	ethanol	−20.51	−18.25	−0.40
	1-propanol	−20.30	−21.01	0.12
	1-butanol	−19.76	−23.90	0.73
Ethers	methyl propyl ether	−6.95	−15.20	1.44
	tetrahydrofuran	−13.06	−16.45	0.59
	ethyl phenyl ether	−17.92	−23.65	1.00
Aldehydes	propanal	−14.40	−17.29	0.51
	butanal	−13.31	−19.34	1.06
	benzaldehyde	−16.83	−25.66	1.55
Ketones	acetone	−15.91	−13.19	−0.48
	2-butanone	−15.53	−15.82	0.05
	acetophenone	−19.17	−28.21	1.58

Esters	methyl formate	−11.64	−11.80	0.03
	methyl acetate	−13.86	−14.82	0.17
	methyl benzoate	−17.92	−30.39	2.19
Amines	ethylamine	−19.30	−17.12	−0.38
	butylamine	−18.33	−22.40	0.71
	aniline	−22.98	−28.09	0.89
Nitrogen with heteroring	pyridine	−19.63	−22.35	0.48
	2-methlypyridine2-	−19.34	−25.70	1.11
	2-methylpyrazine	−23.07	−24.57	0.26
Nitriles	acetonitrile	−16.28	−13.19	−0.54
	butyronitrile	−15.53	−16.45	0.16
	benzonitrile	−17.16	−25.49	1.46
Nitro compounds	1-nitropropane	−13.98	−18.59	0.81
	1-nitrobutane	−12.89	−21.39	1.49
	nitrobenzene	−17.25	−27.75	1.84
Thiols	1-propanethiol	−4.40	−14.73	1.81
	thiophenol	−10.67	−25.07	2.52
	thioanisole	−11.43	−27.08	2.74
Organosulfides[2]	dimethyl sulfide	−6.45	−17.75	1.98
	diethyl sulfide	−5.99	−17.12	1.95
	dipropyl sulfide	−5.32	−16.28	1.92
Organophosphate[3]	trimethyl phosphate	−36.42	−32.69	−0.65
	triethyl phosphate	−32.65	−37.17	0.79
	tripropyl phosphate	−25.53	−36.21	1.87

[1] Experimental ΔG_{water} and $\Delta G_{octanol}$ values are cited from a paper by Wang et al.[48] The $\log P_{ow}$ values were calculated from these values using Eq. (6). [2] Compounds with sulfur atoms. [3] Compounds with phosphate atoms.

Table S2. Additional test set.[1]

Compound	$\log P_{ow}$ (Exp.)	Compound	$\log P_{ow}$ (Exp.)
rufinamide	0.84	tafluprost	4.05
tapentadol	2.87	spinosad	5.9
prasugrel	3.54	vismodegib	2.7
mevastatin	3.95	fingolimod	4.18
artemether	3.53	vemurafenib	3.0
desvenlafaxine	2.6	arbaclofen	1.3
methytestosterone	3.36	tofacitinib	1.81
carglumic acid	−1.10	formestane	2.66
chenodeoxycholic acid	4.15		

[1] $\log P_{ow}$ values from a paper by Daina et al.[49]

Table S3. Additional test set for comparing with our results.[1]

Compound	logP_{ow} (Exp.)	Compound	logP_{ow} (Exp.)
methanol	-0.66	2-5-dimethylphenol	2.34
trimethylamine	0.27	3-4-dimethylphenol	2.23
2-2-2-trichloro-1-dimethoxy-phosphorylethanol	0.51	p-ethylphenol	2.5
diethylamine	0.57	coumarin	1.39
pentachlorophenol	5.01	1-3-indanedione	0.36
quinone	0.2	cinnamamide	1.43
p-iodophenol	2.91	2-chloro-1-4-napthoquinone	2.15
o-nitrophenol	1.79	nicotine	1.17
benzaldehyde	1.48	ethyl cinnamate	2.99
salicylic acid	2.26	(4-bromo-benzal)-acetylacetone	2.75
m-methylphenol	1.96	ethyl benzalcyanoacetate	2.43
o-methylphenol	1.95	2-butylthio-1-4-naphthoquinone	3.29
p-methylphenol	1.94	1-cinnamoylpiperidine	2.74
o-toluidine	1.29	erythromycin	2.48
p-toluidine	1.39	2-5-dimethylphenol	2.34
phthalimide	1.15	3-4-dimethylphenol	2.23
vanillin	1.21	p-ethylphenol	2.5
2-4-dimethylphenol	2.35	coumarin	1.39

[1] logP_{ow} values from a paper by Bannan et al.

Table S4. Correlation coefficient of logP_{ow} values using between B3LYP/6-31G* and the different methods and basis sets.[1]

Parameters	HF/6-31G*	MP2/6-31G*	B3LYP/cc-pVTZ	B3LYP/STO-3G
$P_{logP}\{v, \{v, o\}\}$	0.94	0.90	0.99	0.95
$P_{logP}\{o, \{v, o\}\}$	0.96	0.88	0.99	0.94
$P_{logP}\{w, \{v, o\}\}$	0.96	0.88	0.99	0.91
$P_{logP}\{v, \{o, o\}\}$	0.79	0.87	0.97	0.94
$P_{logP}\{o, \{o, o\}\}$	0.86	0.86	0.95	0.92
$P_{logP}\{w, \{o, o\}\}$	0.85	0.86	0.96	0.89
$P_{logP}\{v, \{w, o\}\}$	0.79	0.89	0.99	0.80
$P_{logP}\{o, \{w, o\}\}$	0.86	0.87	1.00	0.76
$P_{logP}\{w, \{w, o\}\}$	0.85	0.88	1.00	0.72

[1] logP_{ow} values were calculated for five compounds (dimethyl sulfide, ethyl phenyl ether, naphthalene, 1-nitropropane and tribromomethane). The λ values in the free energy calculation procedure were used as follows: With λ_{LJ} = 0.0, λ_{C} was increased in steps of 0.1 from 0 to 1, and with λ_{C}=1, λ_{LJ} was increased in steps of 0.1 from 0 to 1. A total of 21 λ-points were considered in these calculations.

Table S5. Summary of logP_{ow} calculations for 17 compounds.

Parameters	R	R^2	RMSE[1] (kJ/mol)	MAE[2] (kJ/mol)
$P_{logP}\{v, \{v, v\}\}$	0.88	0.77	5.12	4.81
$P_{logP}\{v, \{v, o\}\}$	0.89	0.80	5.01	4.77
$P_{logP}\{v, \{v, w\}\}$	0.89	0.80	5.10	4.88
$P_{logP}\{v, \{o, v\}\}$	0.76	0.58	6.05	5.79
$P_{logP}\{v, \{o, o\}\}$	0.71	0.51	6.05	5.82
$P_{logP}\{v, \{o, w\}\}$	0.72	0.51	5.95	5.73
$P_{logP}\{v, \{w, v\}\}$	0.74	0.55	6.16	5.93
$P_{logP}\{v, \{w, o\}\}$	0.67	0.45	6.11	5.88
$P_{logP}\{v, \{w, w\}\}$	0.67	0.45	6.14	5.91
$P_{logP}\{o, \{v, v\}\}$	0.90	0.81	3.25	2.83
$P_{logP}\{o, \{v, o\}\}$	0.90	0.82	3.16	2.78
$P_{logP}\{o, \{v, w\}\}$	0.92	0.85	3.18	2.89
$P_{logP}\{o, \{o, v\}\}$	0.87	0.75	4.00	3.80
$P_{logP}\{o, \{o, o\}\}$	0.83	0.69	4.00	3.83
$P_{logP}\{o, \{o, w\}\}$	0.81	0.66	3.92	3.74
$P_{logP}\{o, \{w, v\}\}$	0.85	0.73	4.10	3.94
$P_{logP}\{o, \{w, o\}\}$	0.80	0.64	4.05	3.90
$P_{logP}\{o, \{w, w\}\}$	0.80	0.64	4.08	3.93
$P_{logP}\{w, \{v, v\}\}$	0.90	0.82	2.68	2.20
$P_{logP}\{w, \{v, o\}\}$	0.90	0.82	2.59	2.16
$P_{logP}\{w, \{v, w\}\}$	0.93	0.86	2.58	2.26
$P_{logP}\{w, \{o, v\}\}$	0.89	0.79	3.35	3.18
$P_{logP}\{w, \{o, o\}\}$	0.85	0.72	3.36	3.21
$P_{logP}\{w, \{o, w\}\}$	0.82	0.68	3.30	3.12
$P_{logP}\{w, \{w, v\}\}$	0.88	0.78	3.45	3.32
$P_{logP}\{w, \{w, o\}\}$	0.82	0.68	3.42	3.27
$P_{logP}\{w, \{w, w\}\}$	0.81	0.66	3.46	3.30

[1] Root mean square error. [2] Mean average error.

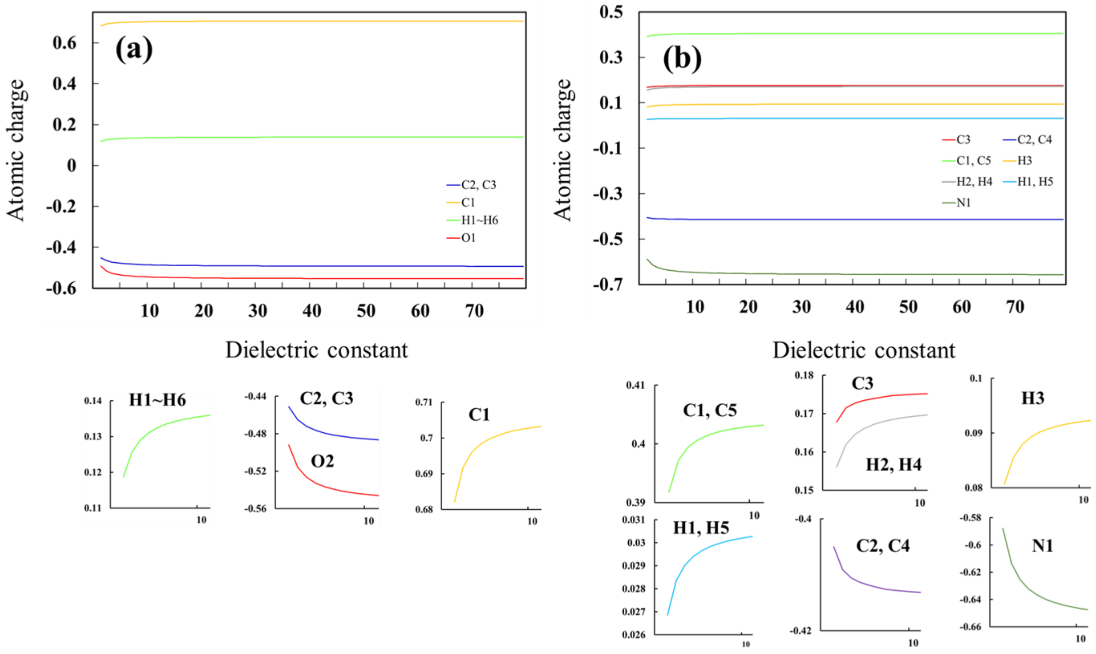

Figure S1. Atomic charges vs. dielectric constant values of (a) Acetone and (b) Pyridine in quantum chemistry calculation. The atomic charges were calculated for dielectric constant increasing every 1. The zooming the graphs of each atom in the range from 0 to 10 dielectric constant are shown at the lower part.

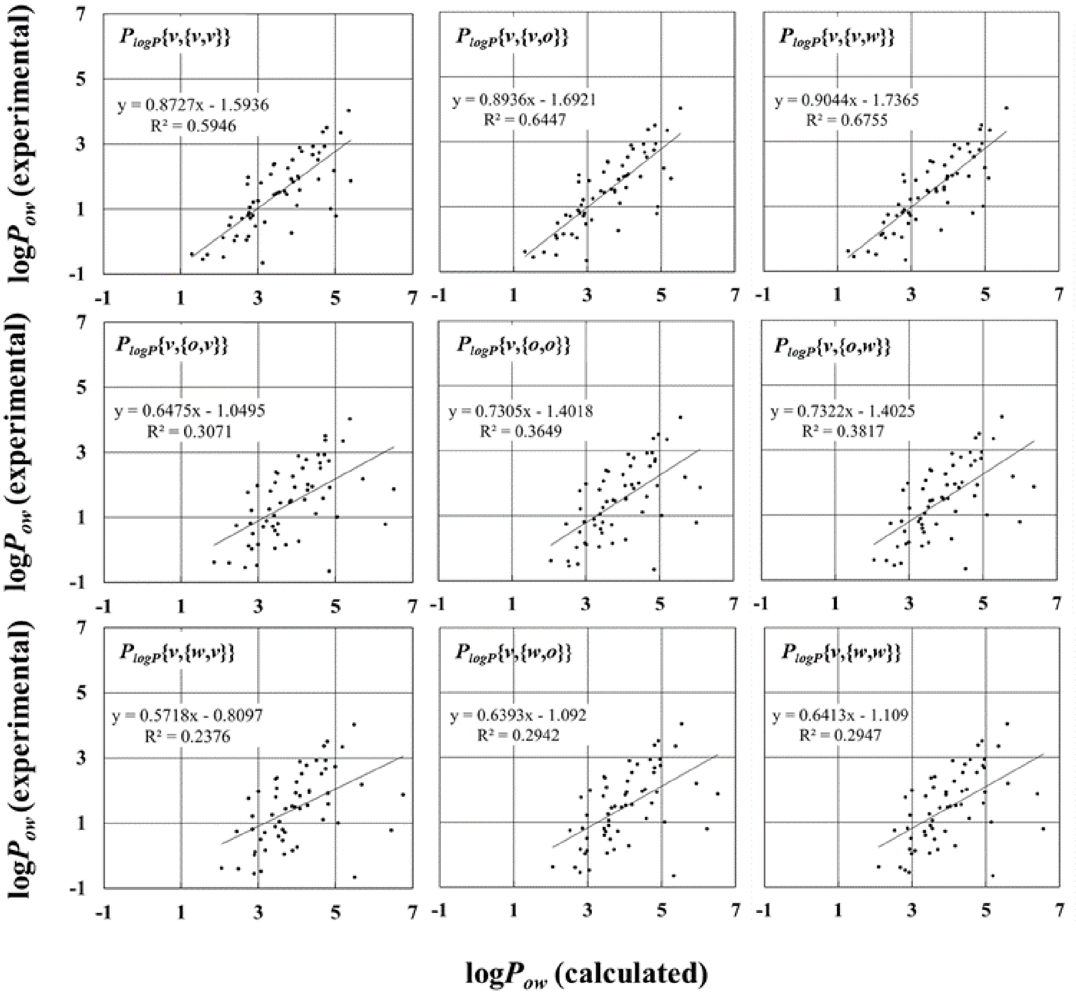

$P_{logP}\{v,\{v,v\}\}$
y = 0.8727x - 1.5936
R² = 0.5946
$P_{logP}\{v,\{v,o\}\}$
y = 0.8936x - 1.6921
R² = 0.6447
$P_{logP}\{v,\{v,w\}\}$
y = 0.9044x - 1.7365
R² = 0.6755
$P_{logP}\{v,\{o,v\}\}$
y = 0.6475x - 1.0495
R² = 0.3071
$P_{logP}\{v,\{o,o\}\}$
y = 0.7305x - 1.4018
R² = 0.3649
$P_{logP}\{v,\{o,w\}\}$
y = 0.7322x - 1.4025
R² = 0.3817
$P_{logP}\{v,\{w,v\}\}$
y = 0.5718x - 0.8097
R² = 0.2376
$P_{logP}\{v,\{w,o\}\}$
y = 0.6393x - 1.092
R² = 0.2942
$P_{logP}\{v,\{w,w\}\}$
y = 0.6413x - 1.109
R² = 0.2947
$\log P_{ow}$ (experimental)
$\log P_{ow}$ (calculated)

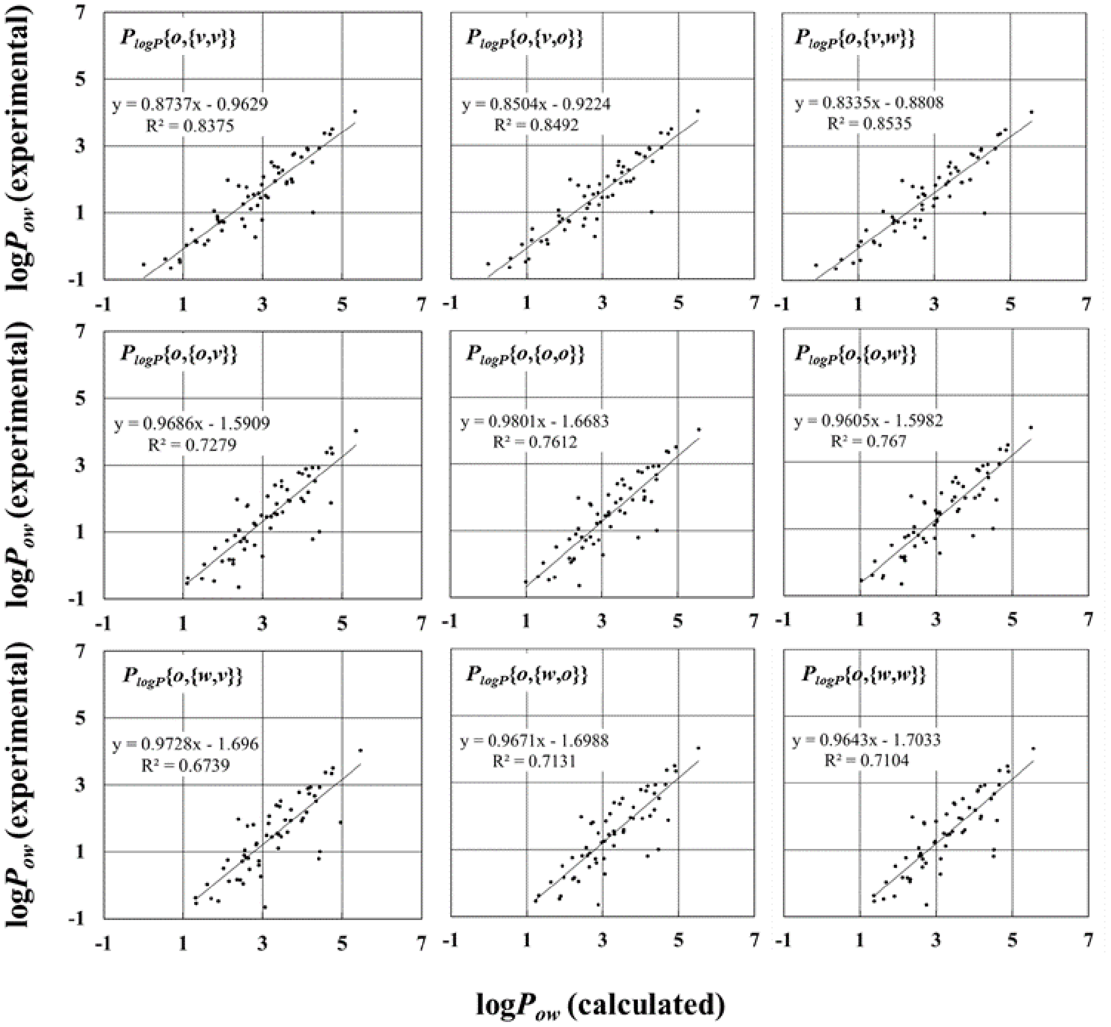

$P_{logP}\{o,\{v,v\}\}$
y = 0.8737x - 0.9629
R² = 0.8375
$P_{logP}\{o,\{v,o\}\}$
y = 0.8504x - 0.9224
R² = 0.8492
$P_{logP}\{o,\{v,w\}\}$
y = 0.8335x - 0.8808
R² = 0.8535
$P_{logP}\{o,\{o,v\}\}$
y = 0.9686x - 1.5909
R² = 0.7279
$P_{logP}\{o,\{o,o\}\}$
y = 0.9801x - 1.6683
R² = 0.7612
$P_{logP}\{o,\{o,w\}\}$
y = 0.9605x - 1.5982
R² = 0.767
$P_{logP}\{o,\{w,v\}\}$
y = 0.9728x - 1.696
R² = 0.6739
$P_{logP}\{o,\{w,o\}\}$
y = 0.9671x - 1.6988
R² = 0.7131
$P_{logP}\{o,\{w,w\}\}$
y = 0.9643x - 1.7033
R² = 0.7104
$\log P_{ow}$ (experimental)
$\log P_{ow}$ (calculated)

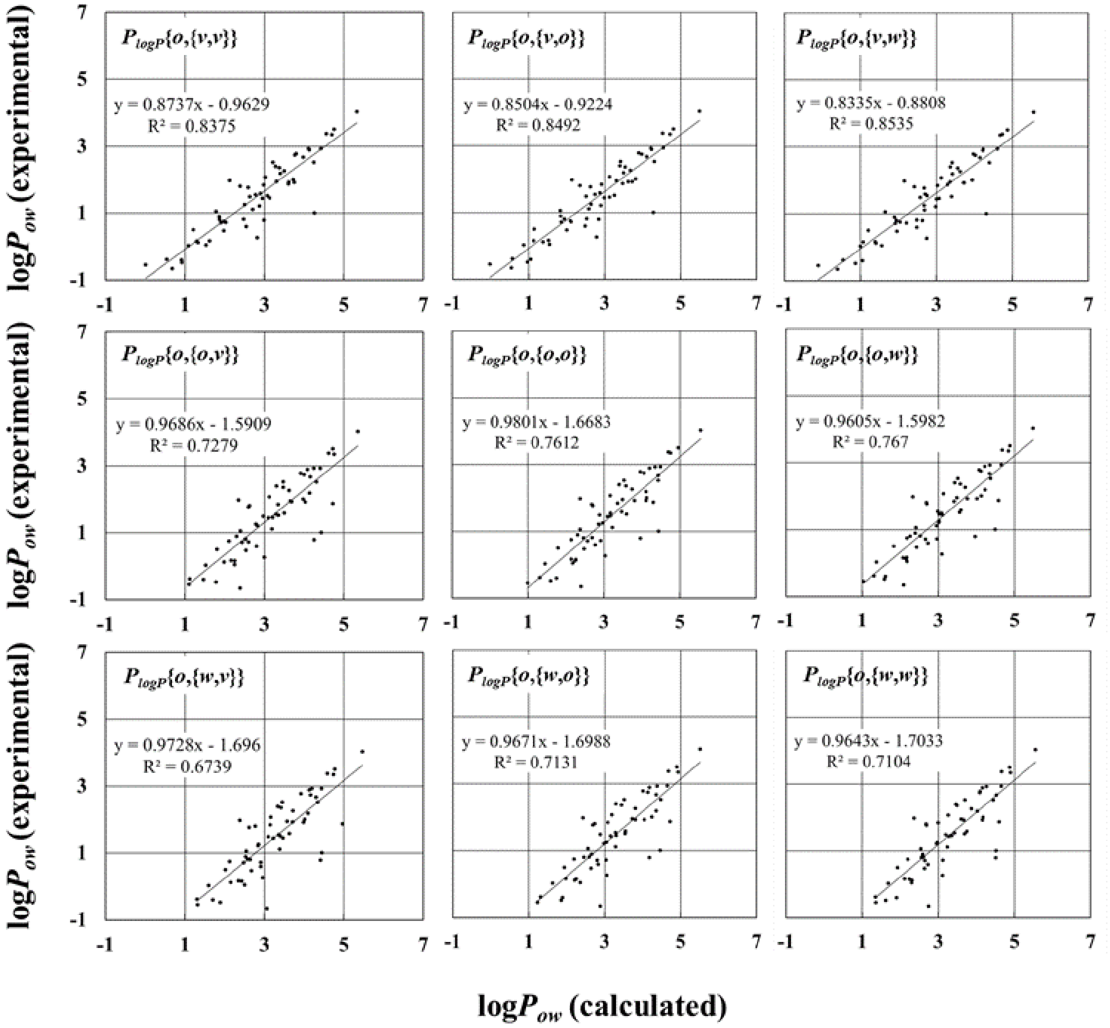

Figure S2. Scatter diagram of calculated and experimentally measured $\log P_{ow}$ values. R^2 and regression line are shown.

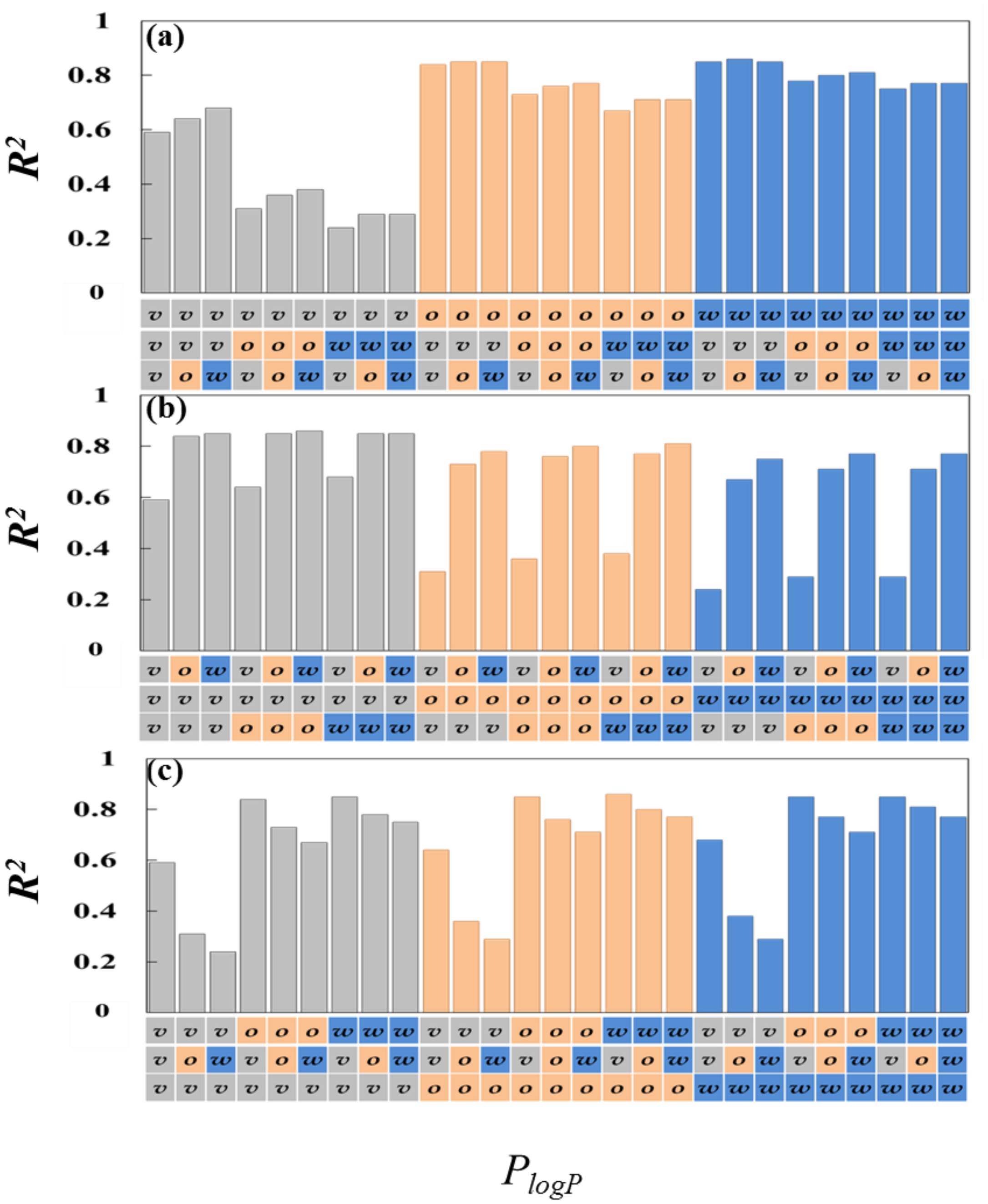

Figure S3. Bar graphs of R^2 values for logP_{ow}. The 1st, 2nd and 3rd lines of the x axis correspond to the atomic charges of compound in the ΔG_{water} calculations, compound in the $\Delta G_{octanol}$ calculations and octanol in the $\Delta G_{octanol}$ calculations, respectively. The three graphs show the same R^2 values, but different orders of P_{logP} arguments. .

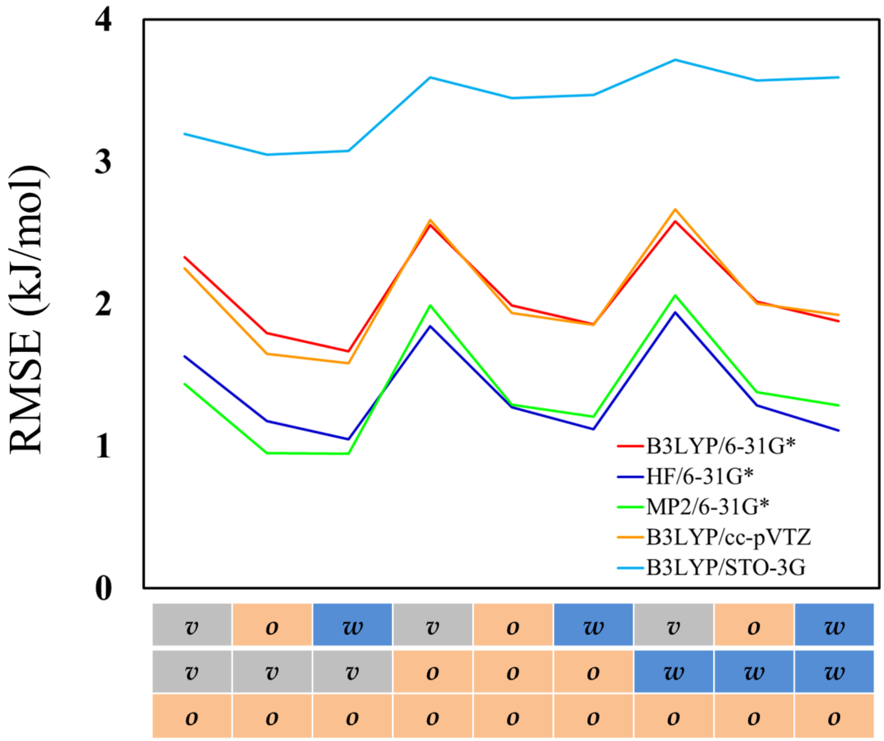

Figure S4. RMSE of logP_{ow} for five compounds (dimethyl sulfide, ethyl phenyl ether, naphthalene, 1-nitropropane and tribromomethane) calculated using different methods and basis sets.
